# Use of Nonvascularized Structural Bone Allografts for Reconstruction of Massive Post-Traumatic Segmental Femoral Bone Defects: A Retrospective Case Series

**DOI:** 10.3390/jcm15135266

**Published:** 2026-07-06

**Authors:** Arpad Solyom, Mihai Mathe, Fodor Pal

**Affiliations:** Faculty of Medicine, “George Emil Palade” University of Medicine, Pharmacy, Sciences and Technology, Street Gheorghe Marinescu 38, 540139 Targu Mures, Romania; solyomarpad@yahoo.com (A.S.); pal.fodor@umfst.ro (F.P.)

**Keywords:** massive bone defect, femoral reconstruction, high-energy trauma, intramedullary nailing, open fracture, structural bone allograft, limb salvage

## Abstract

**Background:** Massive post-traumatic segmental bone defects exceeding 5 cm—particularly those resulting from Gustilo–Anderson type IIIB open fractures—present a formidable reconstructive challenge. Autologous bone graft, though biologically superior, is restricted in this context by limited harvest volume and donor-site morbidity. Nonvascularized structural bone allografts provide unrestricted graft volume and immediate mechanical support, but data on their performance in severely compromised post-traumatic environments remain sparse. **Methods:** This retrospective case series evaluated three patients with massive distal femoral bone loss following high-energy trauma. All were managed with a staged protocol: radical debridement and temporary joint-spanning external fixation at index surgery, followed by definitive reconstruction with a matched nonvascularized structural allograft and retrograde intramedullary nailing once the host bed was aseptic and soft-tissue coverage was established. **Results**: Limb salvage was achieved in all three patients at a mean follow-up of 18 months. Two patients attained stable biological integration and restored anatomical alignment; one of these progressed to full unassisted weight-bearing by four months postoperatively. The third patient developed symptomatic pseudarthrosis at the distal host–graft junction. The proximally integrated allograft had preserved sufficient femoral bone stock to serve as anchorage for a segmental total knee arthroplasty performed two years after the index reconstruction, ultimately preventing amputation. **Conclusions**: Nonvascularized structural allografts combined with rigid intramedullary nailing constitute a reproducible limb salvage strategy for massive femoral defects. The allograft functions not only as a vehicle for primary biological union, but as a structural reserve—maintaining femoral axis and bone stock that remains available for secondary arthroplasty reconstruction should union fail.

## 1. Introduction

Massive segmental bone defects following high-energy trauma—particularly Gustilo–Anderson type IIIB open fractures—continue to pose one of the most demanding challenges in limb salvage surgery. The combination of severe soft-tissue destruction, heavy contamination, and extensive osseous loss leaves surgeons with the difficult task of restoring both length and alignment in an already compromised biological environment [1,2]. Critical-sized defects, generally accepted as exceeding 3–5 cm or approximately twice the diaphyseal diameter, do not heal spontaneously. Left without aggressive reconstruction, these patients are at substantial risk of nonunion, chronic infection, or eventual amputation [3].

Autologous bone graft remains the biological reference standard given its osteogenic and osteoinductive properties, yet its use becomes impractical in the setting of massive defects. Harvest volumes are inherently limited, and obtaining large quantities of autograft carries significant donor-site morbidity [4,5].

Several reconstructive strategies have therefore been developed to address this problem. The Masquelet induced-membrane technique, vascularized fibular autografts, and distraction osteogenesis each represent established options [6]. While distraction osteogenesis with Ilizarov frames has shown long-term success and functional viability in the management of complex tibial fractures and deformities [7], the technique demands prolonged external fixation—often one to two months per centimeter of regenerated bone. Furthermore, it is frequently associated with pin-tract infections, patient non-compliance, and coronal plane deformity [8]. Similarly, the Masquelet technique has gained widespread adoption for diaphyseal defects [9,10], though its outcomes in gaps exceeding 10 cm are less consistent, with spacer failure and late graft resorption representing recognized concerns [11]. Vascularized fibular transfer is effective but requires microsurgical expertise and extended operative time, limiting its applicability in polytrauma patients.

Nonvascularized structural bone allografts offer a pragmatic alternative for large metaphyseal and diaphyseal gaps. They provide immediate mechanical support and essentially unlimited graft volume without donor-site morbidity, while acting as an osteoconductive scaffold for progressive creeping substitution [12]. Their principal limitations are well documented—delayed biological incorporation, susceptibility to deep infection, and late mechanical failure through fracture or collapse—all of which are further amplified in a post-traumatic, poorly vascularized host bed.

The distal femur presents additional biomechanical complexity. It is subject to substantial bending and shear forces during weight-bearing, the metaphyseal flare complicates stable fixation, and its proximity to the knee joint means that even modest malalignment translates into meaningful functional consequences [13]. In this region, spanning the allograft with an intramedullary nail creates a load-sharing construct that protects the graft from early mechanical failure while the surrounding soft tissues recover [14].

Despite a growing body of literature on bone substitutes in orthopedic surgery, outcome data specific to structural allografts used in massive, infection-prone distal femoral defects remain scarce. This study was undertaken to address that gap. The primary objective was to evaluate the clinical and radiographic outcomes of reconstructing massive (>5 cm) distal femoral defects with nonvascularized structural bone allografts stabilized by intramedullary fixation. Secondary objectives included characterizing the postoperative course, assessing the viability of the reconstructed segment as a platform for subsequent arthroplasty, and describing any complications observed associated with this approach.

## 2. Materials and Methods

### 2.1. Study Design and Participants

This retrospective case series included patients treated for massive post-traumatic distal femoral bone defects at the 2nd Orthopedics and Traumatology Clinic, Emergency Clinical County Hospital, Târgu Mureș, Romania, between 2022 and 2025. The study was conducted in accordance with the principles of the Declaration of Helsinki and approved by the Institutional Ethics Committee of “Spitalul Clinic Judetean de Urgenta Targu-Mures” (protocol code: F-PS-0113-07; date of approval: 4 February 2026) for studies involving humans.

Patients were eligible for inclusion based on the following criteria: (1) age 30 to 55 years; (2) massive segmental bone loss of the distal femur (>5 cm) secondary to high-energy trauma; (3) Gustilo–Anderson type IIIB soft-tissue injury; and (4) definitive reconstruction with a nonvascularized structural bone allograft combined with intramedullary nailing.

The 30-to-55 age bracket reflects the demographic characteristics of the patients included in this particular case series—active individuals with favorable biological healing potential and high mechanical demands—rather than representing a strict biological threshold for the procedure itself. Patients with active uncontrolled infection at the time of planned reconstruction, or with systemic conditions precluding major surgery, were excluded. Three patients met all inclusion criteria and formed the study cohort.

### 2.2. Surgical Protocol

Reconstruction followed a strict staged protocol addressing both the biological and mechanical dimensions of limb failure.

#### 2.2.1. Stage 1: Damage Control and Acute Management

The index procedure consisted of radical debridement of all nonviable bone and necrotic soft tissue, followed by high-volume pulsatile lavage. Provisional stability was achieved with a joint-spanning external fixator (DePuy Synthes, West Chester, PA, USA). Deep tissue cultures were obtained intraoperatively to direct targeted intravenous antibiotic therapy. Soft-tissue defects were managed in collaboration with the plastic surgery team; negative-pressure wound therapy (NPWT) (V.A.C.® Therapy System, KCI/3M, San Antonio, TX, USA) and dermal grafts were used as needed to establish a sealed, well-vascularized envelope.

#### 2.2.2. Stage 2: Defect Preparation and Allograft Implantation

Definitive reconstruction was undertaken only after confirmed eradication of infection, defined as normalization of inflammatory markers (CRP and ESR) and negative deep tissue cultures, as well as adequate healing of the soft-tissue envelope. The interval from injury to reconstruction ranged from 1 to 2 weeks.

Following external fixator removal, the fibrotic recipient bone ends were debrided to healthy bleeding cortex—the “paprika sign”—to optimize healing potential at the host–graft junctions. Structural femoral allografts were fresh-frozen, sourced from a tissue bank (Banca de Țesuturi, Spitalul Clinic Județean de Urgență Târgu Mureș, Târgu Mureș, Romania) in our county, and stored at −80 °C. Intraoperatively, each allograft was thawed in warm saline, sized to match the defect dimensions, and impacted into the recipient site.

#### 2.2.3. Stage 3: Definitive Fixation

The entire host–graft–host construct was stabilized with a retrograde intramedullary nail (Expert Retrograde Femoral Nail, DePuy Synthes, West Chester, PA, USA). The host canal was reamed, and multiple interlocking screws were placed proximally and distally to ensure rotational and axial stability. This configuration allowed the nail to function as a load-sharing device, protecting the allograft throughout the incorporation period.

### 2.3. Postoperative Protocol and Outcome Measures

All limbs were immobilized postoperatively in a hinged knee brace (DonJoy, DJO Global, Vista, CA, USA) locked in full extension. Strict non-weight-bearing was maintained for 4 weeks; progressive partial weight-bearing was permitted only upon radiographic evidence of early callus formation at the host–graft junctions.

Follow-up assessments were conducted at 6 weeks, 3 months, 6 months, 12 months, and annually thereafter. Primary endpoints were restoration of limb alignment, full painless weight-bearing, and radiographic graft integration, defined as bridging callus across at least three of four cortices on standard anteroposterior and lateral radiographs. Secondary endpoints included construct survivorship and complication rates—specifically deep infection, nonunion, graft resorption, implant failure, and conversion to total knee arthroplasty. Clinical data were collected and tabulated using Microsoft Excel (Microsoft Corporation, Redmond, WA, USA)

### 2.4. Statistical Analysis

Given the restricted sample size inherent to this specific and highly complex trauma population (*n* = 3), formal inferential statistical analysis was neither feasible nor scientifically appropriate. Consequently, data are presented as a descriptive clinical case series, detailing individual patient trajectories, clinical parameters, and discrete outcomes.

## 3. Results

The study included three patients (two females, one male) with a mean follow-up of 18 months following definitive reconstruction. Patient demographics, injury characteristics, and surgical outcomes are summarized in Table 1.

The staged damage-control approach successfully prevented deep intraosseous infection in all three cases—the most feared early complication in severe open injuries of this type. Joint-spanning external fixation provided adequate skeletal stability during the soft-tissue optimization phase, allowing the plastic surgery team to complete wound coverage and establish a sealed, well-vascularized envelope prior to definitive reconstruction.

Implantation of the structural allografts with retrograde intramedullary nailing restored leg length and gross anatomical alignment intraoperatively in all cases. Postoperative trajectories, however, differed substantially between patients.

### 3.1. Case 1

Initial diagnosis and clinical context: A 40-year-old woman presented following high-energy trauma with a comminuted segmental distal femur fracture and more than 5 cm of bone loss. The injury involved severe soft-tissue stripping requiring revascularization and dermal grafting.

Chronology and indications:

Stage 1 (Damage Control): Radical debridement was performed and a temporary external fixator was applied to stabilize the limb while the plastic surgery team completed soft-tissue coverage.

Stage 2 (Definitive Reconstruction): Following wound healing and infection clearance, the external fixator was removed. A nonvascularized structural allograft was implanted over a retrograde intramedullary nail to restore mechanical continuity and bone stock.

Complications: None. No intraoperative incidents occurred, and no wound breakdown or deep infection developed between staged procedures.

Radiographic evolution: By six months, radiographs demonstrated progressive incorporation at both the proximal and distal host–graft junctions.

Final outcome: At 18 months postoperatively, the construct remained stable with no evidence of graft resorption or hardware failure. The patient was fully weight-bearing with preserved anatomical alignment, 100° knee flexion, and a 5° extension lag.

### 3.2. Case 2

Initial diagnosis and clinical context: In 2016, a 30-year-old man sustained a Gustilo–Anderson type IIIB open distal femur fracture with a segmental bone defect exceeding 5 cm following high-energy trauma.

**Figure 1:**
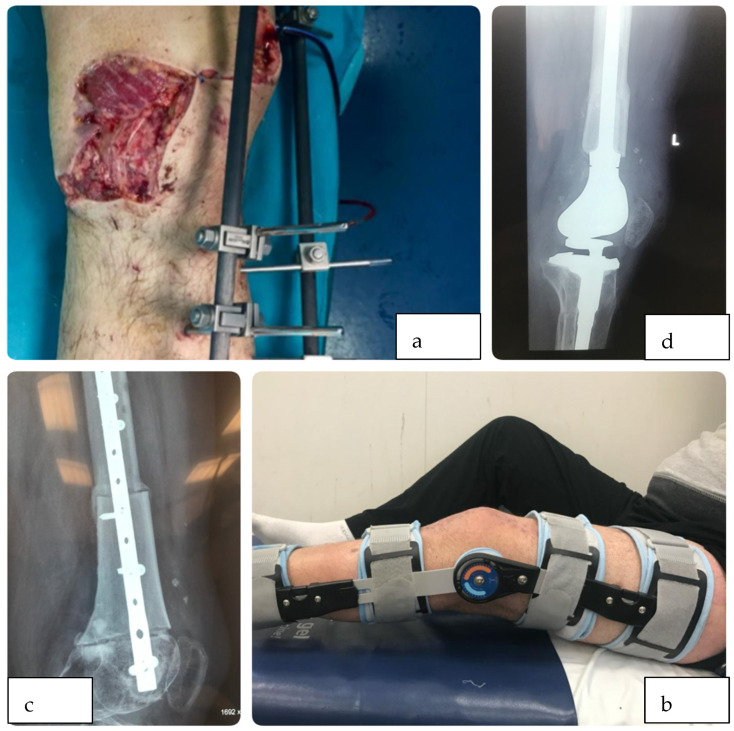
Case 2: Postoperative course of a 30-year-old male with a massive defect, complicated by nonunion and ultimately treated with conversion to arthroplasty. (**a**) Clinical presentation of the massive bone defect and soft-tissue loss managed with a temporary joint-spanning external fixator during the initial damage-control surgery in July 2016. (**b**) Clinical photograph showing the patient fitted with a hinged knee brace following external fixator removal to allow for early joint mobility. (**c**) Radiograph demonstrating the third surgery with an intramedullary nail and structural allograft. This intermediate stage ultimately failed to achieve distal union, developing a symptomatic pseudarthrosis. (**d**) Final anteroposterior radiograph demonstrating successful conversion to a total segmental knee arthroplasty, utilizing the proximally integrated allograft for stable stem anchorage in July 2018.

Chronology and indications:

Stage 1: Index surgery consisted of aggressive debridement and application of a joint-spanning external fixator to protect the soft-tissue envelope and maintain skeletal alignment (Figure 1a). Following external fixator removal, the patient was fitted with a hinged knee brace to allow for early joint mobility (Figure 1b).

Stage 2: After soft-tissue recovery, definitive reconstruction was performed using a structural allograft and an intramedullary nail (Figure 1c).

Complications: Both procedures were uneventful intraoperatively. Subsequently, the patient developed symptomatic nonunion at the distal host–graft junction, attributed to severely compromised local vascularity from the initial injury.

Radiographic evolution: Despite distal pseudarthrosis, the proximal portion of the allograft achieved solid integration into the native host femur.

Final outcome: Two years post-reconstruction (2018), the distal nonunion necessitated conversion to total segmental knee arthroplasty (Figure 1d). The well-integrated proximal allograft provided adequate bone stock for stable stem anchorage. At final follow-up—more than two years after the salvage procedure—the limb remained functional and amputation was avoided.

### 3.3. Case 3

**Figure 2:**
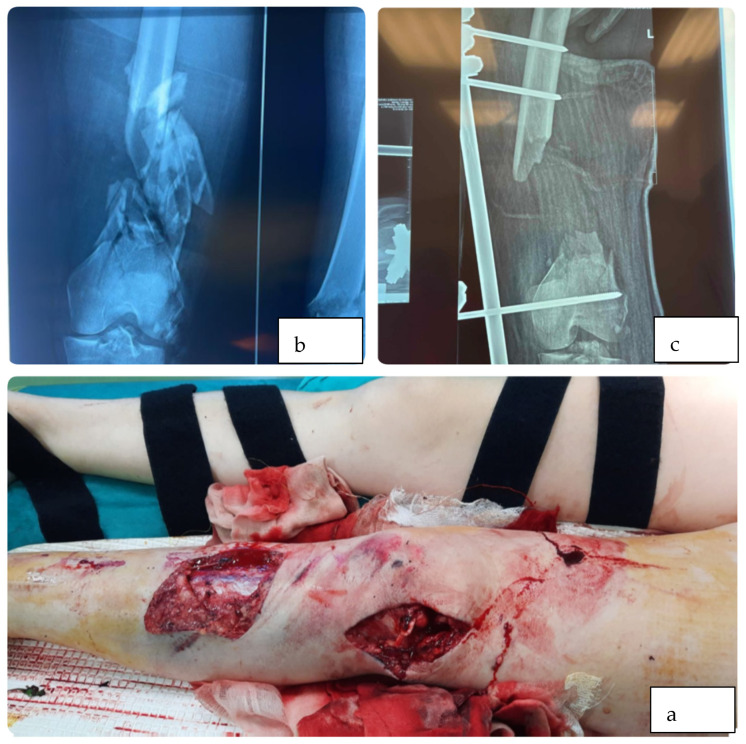
Case 3 (Part 1): Initial presentation and damage-control phase in a 50-year-old female following high-energy trauma. (**a**) Clinical presentation of the massive Gustilo–Anderson type IIIB open fracture in June 2022. (**b**) Initial anteroposterior radiograph revealing the severe comminuted distal femoral fracture with massive segmental bone loss in June 2022. (**c**) Radiograph demonstrating the first-line stabilization utilizing a joint-spanning external fixator to allow for soft-tissue management in July 2022.

Initial diagnosis and clinical context: A 50-year-old woman presented in June 2022 following a motorcycle accident with a Gustilo–Anderson type IIIB open fracture. Admission radiographs demonstrated a severely comminuted distal femur with extensive segmental bone loss (Figure 2a) and a substantial soft-tissue defect. (Figure 2b).

Chronology and indications:

Stage 1: Damage control consisted of radical local debridement. In July 2022, a joint-spanning external fixator was applied to maintain alignment during soft-tissue recovery (Figure 2c).

**Figure 3:**
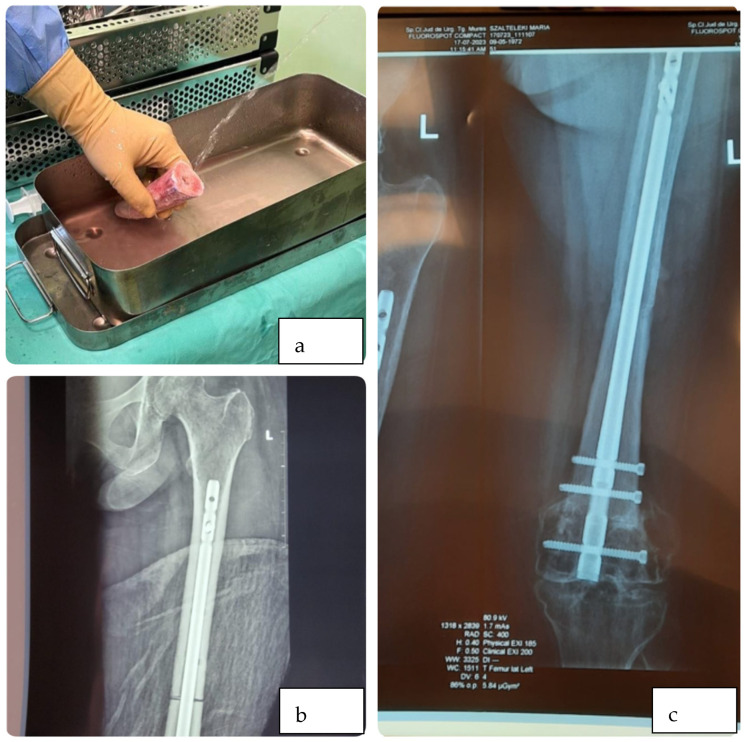
Case 3 (Part 2): Definitive reconstruction and progressive graft integration. (**a**) Intraoperative preparation and thawing of the structural nonvascularized bone allograft in warm saline in October 2022. (**b**) Postoperative radiograph showing the definitive construct stabilized with a retrograde intramedullary nail in October 2022. (**c**) Final follow-up radiograph demonstrating solid bridging callus and successful biological integration of the bone graft in February 2023.

Stage 2: By October 2022, the wound bed had healed sufficiently to proceed with definitive reconstruction using a nonvascularized structural allograft and an intramedullary nail (Figure 3a,b).

Complications: No intraoperative complications occurred. Staged management successfully prevented deep osseous infection.

Radiographic evolution: Bridging callus was visible on standard radiographs four months after reconstruction, representing rapid graft integration for a defect of this magnitude.

Final outcome: By February 2023, the graft was solidly incorporated (Figure 3c). The patient was fully weight-bearing without assistance and had recovered a functional range of motion, with 100° knee flexion and a 5° extension lag.

## 4. Discussion

Limb salvage following Gustilo–Anderson type IIIB open fractures with massive distal femoral bone loss represents one of the most demanding reconstructive problems in orthopedic trauma surgery. The findings of this case series support the use of a strictly staged protocol—prioritizing infection eradication and soft-tissue coverage before structural allografting and intramedullary fixation—as a viable pathway to restore limb continuity in this setting.

Massive nonvascularized structural allografts function exclusively as osteoconductive scaffolds. They carry no intrinsic osteogenic potential and depend entirely on creeping substitution from the host bed [15]. Given the slow, years-long time course of this remodeling process, primary mechanical stability is the dominant determinant of outcome. Rigid interlocking intramedullary fixation is therefore not optional; it neutralizes the shear and rotational forces acting at the host–graft interfaces and establishes the mechanical conditions necessary for neoangiogenesis and osteon bridging to proceed.

Comparison with the principal alternatives clarifies the role of this approach. The Masquelet technique is well suited to infection management and bone regeneration, but defects of this magnitude typically require large volumes of autologous cancellous graft—often necessitating RIA harvest from additional skeletal sites, with attendant morbidity—and the technique does not provide immediate mechanical integrity in a major weight-bearing segment [16]. Distraction osteogenesis with Ilizarov or hexapod frames offers sound biological principles and has demonstrated successful long-term functional outcomes in complex lower extremity trauma, particularly in comminuted tibial fractures and deformities [7]. However, when applied to the distal femur, prolonged external fixation carries a well-documented burden of pin-tract infections, progressive knee stiffness, and patient non-compliance [17]. Structural allografting avoids these sequelae by restoring anatomy immediately and allowing earlier frame removal.

The long-term vulnerability of massive nonvascularized allografts must nonetheless be acknowledged. Their sustained avascularity and slow remodeling rate render them susceptible to nonunion, fatigue fracture, and late resorption over extended follow-up [18,19]. To reduce these risks—particularly the occurrence of delayed union or distal pseudarthrosis—future protocols may benefit from adjuvant modalities such as pulsed electromagnetic field therapy, low-intensity pulsed ultrasound (LIPUS), or extracorporeal shockwave therapy (ESWT). Individual patient factors also warrant attention. Correcting vitamin D and calcium deficiencies, promoting smoking cessation, and closely supervising weight-bearing progression during rehabilitation, while not strict prerequisites, can serve as valuable adjunctive measures.

### 4.1. Case 1: The Importance of Soft-Tissue Optimization

In this patient, massive bone loss was compounded by extensive soft-tissue destruction requiring revascularization and dermal grafting. This determined the staged approach. Proceeding to bone reconstruction beneath a compromised envelope carries a prohibitive risk of deep infection; accordingly, the joint-spanning external fixator was applied first, providing the mechanical stability necessary for reliable soft-tissue coverage. Definitive allografting was deferred until the dermal graft was fully healed. The radiographic finding of solid host–graft integration at six months supports the principle that soft-tissue optimization, rather than early skeletal reconstruction, establishes the biological conditions under which allograft incorporation can occur.

### 4.2. Case 2: Allograft as a Bone Stock Reserve for Secondary Arthroplasty

Case 2 illustrates a recognized limitation of this technique. Despite adherence to the same staged protocol, the reconstruction failed at the distal host–graft junction due to pseudarthrosis—a complication directly attributable to the severely attenuated local vascularity in the post-traumatic bed.

The overall limb salvage strategy nonetheless succeeded. The two-year interval before salvage surgery allowed the proximal allograft segment to achieve solid biological integration, preserving the femoral axis and sufficient bone stock for stable endoprosthesis anchorage—a principle well established in the oncological and revision arthroplasty literature [20,21]. This case underscores that structural allografts in complex trauma serve a dual purpose: they provide a substrate for primary biological union, and they function as a bone stock reserve that remains available for arthroplasty conversion should union ultimately fail.

### 4.3. Case 3: Favorable Biology and Rapid Incorporation

The high-energy mechanism in this case mandated a damage-control approach, with definitive reconstruction deferred from July to October to allow full normalization of local and systemic inflammatory markers. The subsequent course was notable: bridging callus was evident on radiographs at four months postoperatively, an accelerated timeline relative to the magnitude of the defect. This likely reflects the combined effect of meticulous debridement to healthy bleeding cortex and rigid intramedullary fixation, which neutralized shear forces at the host–graft interfaces and provided the mechanical conditions necessary for creeping substitution and early neoangiogenesis to proceed.

Future investigation should prioritize multicenter prospective registries capable of accumulating sufficient case numbers for meaningful analysis. On the biological side, augmentation of structural allografts with orthobiologics—including bone marrow aspirate concentrate (BMAC), platelet-rich plasma (PRP), or antibiotic-loaded hydrogels—warrants prospective evaluation to determine whether such adjuncts can accelerate neoangiogenesis, reduce late pseudarthrosis rates, and provide early protection against biofilm formation [22].

### 4.4. Study Limitations

The limitations of this study are inherent to its retrospective design and the extremely small sample size (*n* = 3), which precludes broad generalizations. The heterogeneity in patient presentation, varying intervals to definitive reconstruction, and diverse soft-tissue management strategies introduce confounding variables. Furthermore, the absence of a control group limits direct comparative efficacy analysis against other reconstructive modalities. Longer-term follow-up (5–10 years) in larger cohorts is required to accurately quantify the late rates of graft decimation, hardware failure, and secondary osteoarthritis.

## 5. Conclusions

Patients were carefully selected—strictly defined by the absence of active infection, adequate local vascular status, a successfully reconstructed soft-tissue envelope, the absence of severe systemic comorbidities, expected patient compliance with prolonged rehabilitation, and the ability to achieve immediate intraoperative mechanical stability; nonvascularized structural bone allografts combined with intramedullary nailing represent a viable and reproducible strategy for reconstructing massive post-traumatic femoral defects. The efficacy of this approach critically depends on strict adherence to a staged protocol: infection eradication and establishment of a durable soft-tissue envelope are prerequisites, not adjuncts, to graft implantation.

Achieving complete biological union remains the primary clinical challenge. However, the functional value of the structural allograft extends beyond union alone. It restores limb length immediately, provides mechanical continuity, and—crucially—preserves bone stock that remains available for secondary reconstruction should the primary procedure fail. As demonstrated in this series, even a graft that does not achieve full incorporation can serve as a stable biological foundation for subsequent segmental endoprosthetic replacement, avoiding amputation in a patient who would otherwise have few remaining options.

This technique demands rigorous patient selection and sustained long-term follow-up. Within those boundaries, it offers a reliable limb salvage pathway in a clinical scenario where the alternative is frequently loss of the limb.

## Figures and Tables

**Table 1:** Patient demographics, injury characteristics, and clinical outcomes.

Patient	Age/Sex	Mechanism of Injury	Fracture Classification	Soft-Tissue Management	Follow-Up	Complications	Final Clinical Outcome
Case 1	40/F	High-energy trauma	Comminuted segmental > 5 cm	Dermal graft, revascularization	1.5 years	None	Good integration, restored alignment
Case 2	30/M	High-energy trauma	Gustilo–Anderson IIIB > 5 cm	Local coverage	>2 years	Pseudarthrosis	Converted to total segmental TKA (2018)
Case 3	50/F	Motorcycle accident	Gustilo–Anderson IIIB > 5 cm	Wound correction	1.5 years	None	Full weight-bearing at 4 months

## Data Availability

Data are contained within the article.

## Abbreviations

The following abbreviations are used in this manuscript:

BMAC	Bone Marrow Aspirate Concentrate
CRP	C-Reactive Protein
ESR	Erythrocyte Sedimentation Rate
NPWT	Negative-Pressure Wound Therapy
PRP	Platelet-Rich Plasma
RIA	Reamer–Irrigator–Aspirator
